# Factors influencing urban Ghanaian consumers’ preferences for meals/products from multinational food corporations and gender subgroups: a supervised machine learning MaxDiff designs study

**DOI:** 10.3389/fnut.2026.1729484

**Published:** 2026-01-30

**Authors:** Eric Nyarko, Tina Bartelmeß

**Affiliations:** 1 Department of Statistics and Actuarial Science, School of Physical and Mathematical Sciences, University of Ghana, Accra, Ghana; 2 Faculty of Life Sciences, Food, Nutrition and Health, University of Bayreuth, Kulmbach, Germany

**Keywords:** consumers, Ghana, machine learning models, MaxDiff designs, multinational food corporations

## Abstract

The decision-making process of consumers when choosing meals or products from multinational food corporations is influenced by various factors related to food, personal preferences, and the environment. This study combines five machine learning (ML) models and quantitative MaxDiff designs to predict the factors that influence urban Ghanaian consumers’ preferences for meals/products from these corporations, alongside gender-specific differences in consumer preferences. We utilized data collected in March/April 2023 from a random sample of 200 consumers in the Greater Accra region. All ML models demonstrated similar levels of goodness of fit, but there were slight differences in predictive performance. The Ridge regression model distinguished itself with superior predictive capabilities, although it required a longer fitting time. For all respondents, food quality/packaging emerged as the most critical factor in choosing products or meals, followed by healthiness, taste/flavor, and nutritional value. The subgroup results indicated notable gender-specific differences in consumer food preferences. While female respondents placed greater emphasis on attributes such as aroma/smell, followed by affordability, convenience, accessibility, and familiarity with a meal, male respondents prioritized factors like being high in fiber/roughage, followed by aroma/smell, affordability, and convenience. This finding is crucial as it suggests that dietary interventions could benefit from being tailored to specific gender groups. By integrating multiple ML models and MaxDiff designs, we identified additional significant predictors compared to traditional statistical methods, offering policymakers a deeper understanding of the factors influencing urban Ghanaian consumers’ food preferences for products from multinational food corporations. This understanding supports the development of healthier food policies in Ghana’s evolving food landscape.

## 1 Introduction

Globalization has led to the integration of national economies into the global economy, resulting in an increasing flow of goods, services, capital, and technology across borders (1). This free flow of trade and investment allows multinational food corporations, such as supermarkets, fast-food restaurants, manufacturing and processing firms, and retailers, to dominate global trade and investment. These corporations are progressively entering markets in low-income countries (2, 3), impacting the nutrition transition in those regions (4, 5). Researchers argue that this significant shift in food systems and retail environments in developing countries is influencing consumers’ food choices and dietary habits, often in an undesirable direction toward highly processed, energy-dense foods (3). There is a clear causal link between the consumption of such unhealthy foods and various non-communicable diseases (NCDs), including type 2 diabetes, obesity, and coronary heart disease. Unhealthy dietary habits are a major global risk factor for NCDs, and public health nutrition initiatives can help prevent and address these unhealthy patterns in populations (6, 7).

The increasing prevalence of lifestyle-related diseases underscores the critical role of nutrition in maintaining health and preventing chronic conditions. Although appropriate dietary choices support physiological function and disease prevention, the complexity of food interactions, coupled with variability in individual nutritional requirements, renders food selection a highly personalized and challenging process (8). To better understand consumer behavior, particularly regarding the preference for global food products, more sophisticated surveys are needed (6, 9). However, research has so far paid less attention to the consumer perspective and the demand side in developing countries (5, 9).

Maximum difference scaling, used in this study, is a contemporary method in consumer experiments (10), particularly in food-related research (11). Unlike traditional rating-scale surveys, this approach requires participants to engage more cognitively, thereby enhancing their focus during the choice task (12). Furthermore, the literature has emphasized the advantages of maximum difference scaling over discrete choice experiments (13). An early study employed maximum difference scaling to investigate the factors influencing urban Ghanaian consumers’ preferences for multinational food products (14). However, this study did not explore gender-specific differences in consumer preferences, which is crucial for gaining a deeper understanding of diverse consumer segments and for developing effective dietary behavior change strategies. Furthermore, the study did not incorporate machine learning (ML) methods/modeling, which can identify additional significant factors influencing urban Ghanaian consumers’ preferences compared to traditional statistical methods. Notably, while many statistical and ML methods can be used for both inference and prediction, traditional statistical methods primarily focus on inference, whereas ML emphasizes prediction (15), which makes it particularly advantageous when the primary goal is predictive performance rather than making inferences about the population from which the sample was drawn. ML methods utilize learning algorithms to identify patterns in complex data, making minimal assumptions regarding data-generating processes. This effectiveness persists even in the presence of intricate nonlinear interactions in the data. However, despite valid predictive results, the lack of an explicit model can make it challenging to relate ML outputs to existing knowledge directly. Moreover, as the number of input variables and potential associations among them increases, classical statistical models that capture these relationships tend to become more complex, and the accuracy of statistical inferences may diminish (15, 16).

Many studies have sought to integrate ML algorithms into nutrition sciences (17–19). For example, researchers are increasingly exploring how artificial intelligence (AI) and ML models can identify complex patterns that predict consumers’ food preferences and dietary needs (8, 17, 20–23). While these studies mark significant progress in the field, many do not effectively combine ML with data from controlled statistical experiments. Additionally, traditional statistical methods or models often struggle to handle complex relationships within the data or to learn from extensive food-related information. To address this gap and gain a deeper understanding of diverse consumer segments, the present study integrates multiple ML models, including Ridge regression, Elastic Net, LASSO, a Generalized Regression Model with Pruned Forward Selection, Forward Selection, and quantitative MaxDiff designs. This approach offers a more promising strategy for providing comprehensive, AI/ML-driven insights. By identifying additional significant predictors that enhance the model’s performance, the study convincingly demonstrates the advantages of ML over traditional methods. It shows how ML models can capture complexity, leverage non-linear relationships, and evaluate variable importance more thoroughly, leading to deeper insights into the data and improved predictions.

## 2 Methods

### 2.1 Study design and data collection

This study combines five ML models with quantitative MaxDiff designs (known as machine learning MaxDiff designs) to predict the factors influencing meal and product preferences of urban Ghanaian consumers from multinational food corporations, and to explore gender-specific differences in those preferences. For this cross-sectional study, we determined that a minimum of 77 respondents was required, based on the sample size calculation proposed by Hensher et al. (24). Thus, we considered an anticipated sample size of 200 respondents to be sufficient. We employed a random sampling technique and surveyed 200 consumers in the Greater Accra Region (Accra) in March/April 2023. Respondents were approached at a diverse range of multinational supermarkets and international fast-food restaurants, including Shoprite Holdings Ltd., Barcelos Ghana, PICK ‘N PAY, Burger King, Massmart, Chicken Inn, SPAR, Kentucky Fried Chicken, Melcom Group, Pizza Hut, and Pizza Inn. This comprehensive approach ensured that our data was representative and reliable. To assess preferences, each respondent was presented with 20 scenarios generated from a statistical experimental block design. In this design, each attribute appeared five times across all scenarios, and each scenario contained four attributes, with each attribute appearing once with every other attribute. Here, each attribute has an equal chance of being selected. During the survey, respondents were asked to express their preferences by selecting the most important attribute and the least important attribute when considering the purchase of a product from a multinational food corporation. We collected the data using interviewer-administered questionnaires on paper to ensure the quality of our data. A total of 14 attributes were considered, identified through a comprehensive literature review, consultations with experts, and a focus group discussion involving 10 food industry professionals and 30 potential consumers of multinational food and fast-food products. The attributes included: nutritional content, taste, healthiness, image/desirability, familiarity, preparation time, social influence, quality/packaging, availability, accessibility, affordability, convenience, aroma, texture, and visual esthetics. These attributes reflected both intrinsic product characteristics (e.g., nutrition, taste, fiber content) and extrinsic factors (e.g., price, accessibility, packaging, and social norms).

### 2.2 Data preprocessing and method of analysis

The dataset used in this study was initially examined for missing values using the Explore Missing Values feature in JMP software, which identifies patterns of missing data and facilitates imputation. Given the nature of the data, there were no outliers, and no missing values were reported; therefore, no imputation strategies were necessary. This was possible because an interviewer-administered questionnaire was used during the survey, and enumerators were present to conduct initial checks for any incomplete responses. They encouraged respondents to complete any unfinished sections.

Before applying the five machine learning models—Ridge Regression (25), Elastic Net (26), LASSO (27), a Generalized Regression Model with Pruned Forward Selection, and Forward Selection (28, 29)—the dataset was divided using the 5-fold cross-validation method. The dataset was randomly partitioned into 5 parts. The first 4 sections were used for training, and the 5th section was used for validation. The training data was used to develop the five ML models, while the validation data served to assess their performance. To ensure the robustness of our evaluation, we compared model performance using a range of key metrics, including the corrected Akaike Information Criterion (AICc), Bayesian Information Criterion (BIC), Root Average Squared Error (RASE), negative log-likelihood, and model fitting time. These multiple metrics help identify the best-performing model by balancing fit, complexity, efficiency, and predictive power. It is important to note that the predictor variables in our study were diverse, including attributes such as nutritional content/value, image/desirability, high fiber and roughage, taste/flavor, meal preparation time, familiarity with the meal, healthiness, social factors (e.g., family/friends who eat it), food quality/packaging, availability, accessibility, affordability, convenience, aroma/smell, texture, and visual esthetics. The response variable was treated as continuous using effect coding. Regression analysis was appropriate here because we aim to predict continuous outcomes rather than categorical ones. We also assessed the significance of each utility estimate (*β*) related to the independent variables at *p*-values of 0.001, 0.01, or 0.05, along with 95% confidence intervals (CIs) (28). All analyses utilizing both the complete/full dataset and subgroup data categorized by gender were conducted using JMP Pro (Version 17.0).

## 3 Results

### 3.1 Participant characteristics

Table 1 presents the demographic characteristics of the 200 respondents, categorizing the data by gender and reporting the frequency of product consumption from multinational food corporations during the daytime. Most respondents were female (65.1%), and the majority fell within the 15–29 age range. In contrast, a larger proportion of males (34.4%) fell within the age range of 30–49 years. Among the educational qualifications, a slightly higher percentage of females held a bachelor’s degree (39.6%) compared to males (36.6%). Additionally, there were slightly more single males (71.0%) without children (68.8%) compared to females (64.2%). In terms of consumption, a significant number of females reported regularly consuming products from multinational food corporations, with 39.6% indicating they often do, and another 39.6% stating they do so sometimes.

**Table 1:** Characteristics of study population by gender.

Variable	Category	Female (*n = 106*)	Male (*n = 93*)
Age (years)	15–29	69 (65.1%)	56 (60.2%)
	30–49	35 (33.0%)	32 (34.4%)
	50+	2 (1.9%)	5 (5.4%)
Educational level completed	Bachelors	42 (39.6%)	34 (36.6%)
	Diploma/HND	11 (10.4%)	7 (7.5%)
	Masters	9 (8.5%)	10 (10.8%)
	Ph. D./DrPH	5 (4.7%)	5 (5.4%)
	Primary or less	13 (12.3%)	6 (6.5%)
	Secondary school/SHS/SSS	26 (24.5%)	31 (33.3%)
Marital status	Divorced	6 (5.7%)	2 (2.2%)
	Married	24 (22.6%)	22 (23.7%)
	Single	74 (69.8%)	66 (71.0%)
	Widowed	2 (1.9%)	3 (3.2%)
Family status	1 kid	19 (17.9%)	15 (16.1%)
	2–3 kids	17 (16.0%)	10 (10.8%)
	More than 3 kids	2 (1.9%)	4 (4.3%)
	No kids	68 (64.2%)	64 (68.8%)
Eat products/meals from international manufacturers or restaurant chains during the day	Always	11 (10.4%)	21 (22.6%)
	Never	0 (0.0%)	1 (1.1%)
	Often	42 (39.6%)	34 (36.6%)
	Rarely	11 (10.4%)	14 (15.1%)
	Sometimes	42 (39.6%)	23 (24.7%)

### 3.2 Machine learning model performance evaluations

Table 2 provides the model evaluation information for all five ML models, evaluated using the 5-fold cross-validation method, alongside a statistical method (the traditional maximum difference model) utilized in a prior study (14). All five ML models displayed excellent goodness of fit, achieving the lowest AICc value of 6513.1893 and a BIC value of 6616.1162. In comparison, the statistical method/traditional model had an AICc value of 18258.4200 and a BIC value of 18352.3700. While all ML models exhibited the same goodness of fit (AICc of 6513.1893 and BIC of 6616.1162), they demonstrated minimal variations in predictive performance, with a mean RASE ranging from 0.67365 to 0.67372 and fit times in milliseconds (Range: 531 to 11,877). The trade-off between computational efficiency, goodness of fit, and predictive performance is a crucial consideration in model selection. Among these, the Ridge regression model, despite its longer fit time of 7,243 milliseconds, stood out with the best predictive performance, with a mean RASE of 0.67365. Both the Elastic Net and LASSO models demonstrated identical predictive performance, with a mean RASE of 0.67369. However, Elastic Net had a fit time of 11,228 milliseconds, while the LASSO model took the longest time, completing the fit in 11,877 milliseconds. In summary, the Ridge regression model stands out as the preferred candidate due to its combination of superior computational efficiency and predictive performance, achieving the same goodness of fit as the other ML models relative to the number of parameters. Its robust predictive capabilities justify the additional computational costs, making it the ideal choice for applications that prioritize goodness of fit, predictive performance, and computational efficiency.

**Table 2:** Model performance evaluation.

Model	AICc	BIC	Mean RASE	Elapsed time
LASSO	6513.1893	6616.1162	0.67369	11.8770
Generalized regression (forward selection)	6513.1893	6616.1162	0.67372	0.5310
Generalized regression (pruned forward selection)	6513.1893	6616.1162	0.67372	0.6810
Elastic net	6513.1893	6616.1162	0.67369	11.2280
Ridge^a^	6513.1893	6616.1162	0.67365	7.2430
Maximum difference model^b^	18258.4200	18352.37		

a Signifies the best model.

b Traditional model.

### 3.3 Prediction profilers for each type of machine learning model

**Figure 1:**
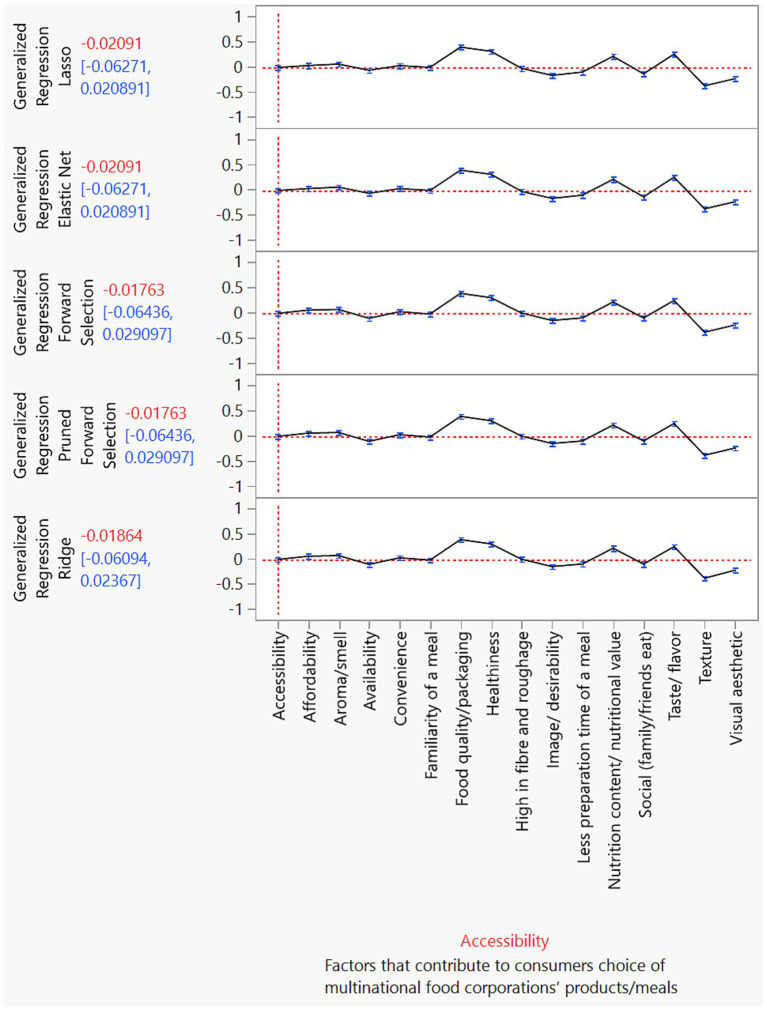
Prediction profiler for each type of machine learning model related to factors that influence consumers’ choice of multinational food corporations’ products/meals. While all models predicted food quality/packaging and healthiness as the most important factors influencing consumer choices of products or meals from multinational food corporations, the texture of a meal was considered less important overall.

Figure 1 illustrates the utility profilers for various ML models based on the full dataset. These profilers highlight the factors influencing consumers’ choices regarding products and meals from multinational food corporations. All ML models consistently identified the following attributes as significant: accessibility, affordability, convenience, familiarity with a meal, food quality/packaging, healthiness, high fiber and roughage content, minimal preparation time, nutritional content/value, social influences (such as family and friends eating), taste, visual appeal, aroma, availability, and image/desirability, and texture. These attributes, identified as significant, provide a comprehensive understanding of consumer behavior in the food industry. However, it is worth noting that the texture of a meal was deemed less necessary overall.

### 3.4 Ridge regression model

The trade-off between computational efficiency, goodness of fit, and predictive performance is a crucial factor in model selection. Consequently, we chose to present the results from the Ridge regression model (see Table 3) because it exhibited the best overall performance among all candidates. As presented in Table 3, all the attributes, according to the full dataset, significantly influenced consumers’ choices regarding products and meals from multinational food corporations. Based on the relative importance of these attributes with reference to the baseline attribute, food quality/packaging produced the highest utility estimate. This finding identifies food quality/packaging as the most significant factor in meal choices, followed by healthiness, taste/flavor, nutrition content/nutritional value, aroma/smell, affordability, convenience, accessibility, high in fiber and roughage, familiarity of a meal, availability, less preparation time of a meal, social (family/friends eat), and image/ desirability. Conversely, although the texture attribute was statistically significant, it was traded off, highlighting the complex decision-making process of consumers.

**Table 3:** Ridge regression model results of factors influencing consumers’ choices of multinational food products.

Attribute	Utility estimate (*β*)	Standard error	Wald ChiSquare	Prob >ChiSquare	Lower 95%	Upper 95%
Accessibility	0.231	0.031	53.1635	<0.0001*	0.1695	0.2942
Affordability	0.2595	0.034	55.2510	<0.0001*	0.1910	0.3279
Aroma/smell	0.2791	0.0322	74.8189	<0.0001*	0.2158	0.3423
Availability	0.1496	0.0329	20.5590	<0.0001*	0.0849	0.214
Convenience	0.2406	0.0335	51.3160	<0.0001*	0.1748	0.3065
Familiarity of a meal	0.1755	0.0328	28.5060	<0.0001*	0.1111	0.2400
Food quality/packaging	0.5931	0.0324	334.1130	<0.0001*	0.5295	0.6567
Healthiness	0.5189	0.0329	248.1891	<0.0001*	0.4544	0.5835
High in fiber and roughage	0.2256	0.0350	41.4404	<0.0001*	0.1569	0.2943
Image/desirability	0.0730	0.0339	4.6172	0.0317*	0.0064	0.1396
Less preparation time of a meal	0.1058	0.0344	9.4269	0.0021*	0.0382	0.1734
Nutrition content/ nutritional value	0.4074	0.0352	133.4735	<0.0001*	0.3383	0.4765
Social (family/friends eat)	0.0952	0.0354	7.2289	0.0072*	0.0258	0.1646
Taste/flavor	0.4552	0.0328	192.4869	<0.0001*	0.3909	0.5195
Texture	−0.1759	0.0328	28.6145	<0.0001*	−0.2403	−0.1114
^a^Visual esthetic						
Model fit statistics						
AICc	6513.1893					
BIC	6616.1162					
Mean RASE	0.6736					
Negative Log-Likelihood	3239.4980					
*P*-value	0.0001					

a Reference/baseline attribute.

AICc, corrected Akaike Information Criterion; BIC, Bayesian Information Criterion; RASE, Root Average Squared Error; * Statistically significant attributes at *p*-values less than 0.001, 0.01, 0.05 or 95% confidence intervals without zero.

### 3.5 Multiple comparisons of attributes based on full dataset

**Figure 2:**
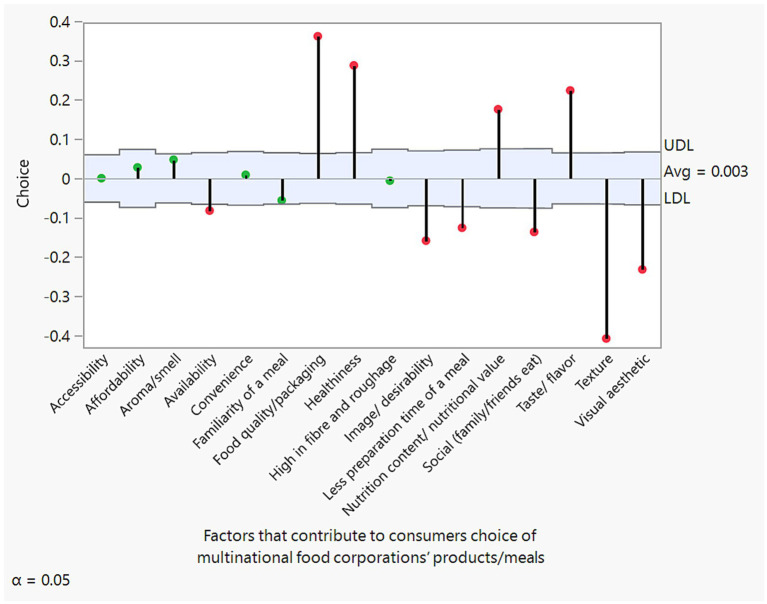
Average decision chart of factors influencing consumers’ choices of multinational food products. Green indicates insignificant factors, while red indicates significant factors.

Figure 2 shows the overall average decision chart. The group average, upper decision limit (UDL), and lower decision limit (LDL) are also presented. An attribute is considered statistically significant if it exceeds either the UDL or the LDL (see Figure 2). With the exceptions of accessibility, affordability, aroma/smell, convenience, familiarity of a meal, and high fiber and roughage—which were found to be statistically insignificant—all other attributes, such as food quality/packaging, healthiness, nutrition content/nutritional value and taste/flavor have significantly positive averages, highlighting their greater preference than the overall average. In contrast, availability, image/desirability, less preparation time of a meal, social (family/friends eat), taste/ flavor, texture, and visual esthetic, show negative average significant differences, indicating their lower preference than the overall average.

### 3.6 Subgroup analysis by gender

The subgroup analyses, comparing the factors influencing consumers’ choices regarding products and meals from multinational food corporations, are presented in Table 4, categorized by male and female respondents in terms of order of importance. For both males and females, food quality/packaging emerged as the most important factor in product/meal choices, followed by healthiness, taste/flavor, and nutritional content/nutritional value. However, female respondents placed higher importance on attributes such as aroma/smell, followed by affordability, convenience, accessibility, and familiarity of a meal in their product/meal choices. In contrast, male respondents placed higher importance on attributes such as high fiber and roughage, followed by aroma/smell, affordability, and convenience. The statistically significant texture attribute, although often traded off, further highlights the complex decision-making process of both male and female consumers in their product/meal choices. Nevertheless, the observed gender-specific preference is crucial for businesses. It can guide targeted marketing efforts and meal/product development strategies to cater to diverse consumer needs, ultimately influencing dietary behaviors and promoting healthier eating habits.

**Table 4:** Ridge regression model results of factors influencing consumers’ choices of multinational food products by gender.

Attribute^f^	Utility estimate (β)^f^	Prob >ChiSquare^f^	Attribute^m^	Utility estimate (β)^m^	Prob >ChiSquare^m^
Food quality/packaging	0.6339	<0.0001*	Food quality/packaging	0.5232	<0.0001*
Healthiness	0.5121	<0.0001*	Healthiness	0.4167	<0.0001*
Taste/flavor	0.4826	<0.0001*	Taste/flavor	0.3625	<0.0001*
Nutrition content/nutritional value	0.4298	<0.0001*	Nutrition content/nutritional value	0.3428	<0.0001*
Aroma/smell	0.3139	<0.0001*	High in fiber and roughage	0.1929	0.0002*
Affordability	0.3095	<0.0001*	Aroma/smell	0.1914	<0.0001*
Convenience	0.2885	<0.0001*	Affordability	0.1538	0.0032*
Accessibility	0.2555	<0.0001*	Convenience	0.1210	0.0169*
Familiarity of a meal	0.2114	<0.0001*	Familiarity of a meal	0.1088	0.0238*
High in fiber and roughage	0.1806	0.0002*	Accessibility	0.0905	0.0573
Availability	0.1424	0.0011*	Availability	0.0501	0.3150
Less preparation time of a meal	0.1221	0.0089*	Less preparation time of a meal	0.0377	0.4626
Social (family/friends eat)	0.0650	0.1724	Social (family/friends eat)	0.0107	0.8328
Image/desirability	0.0483	0.2863	Image/desirability	0.0069	0.8910
Texture	−0.1670	0.0002*	Texture	−0.2337	<0.0001*
^a^Visual esthetic					
Model fit statistics					
AICc	3499.3598		3096.4737		
BIC	3591.4076		3186.2456		
Mean RASE	0.66963		0.67767		
Negative Log-Likelihood	1732.4975		1531.0287		
*P*-value	0.0001		0.0001		

a Reference attribute.

f Results for female participants.

m Results for male participants.

*Statistically significant attributes at *p*-values less than 0.001, 0.01, 0.05.

### 3.7 Multiple comparisons of attributes based on gender-subgroup

**Figure 3:**
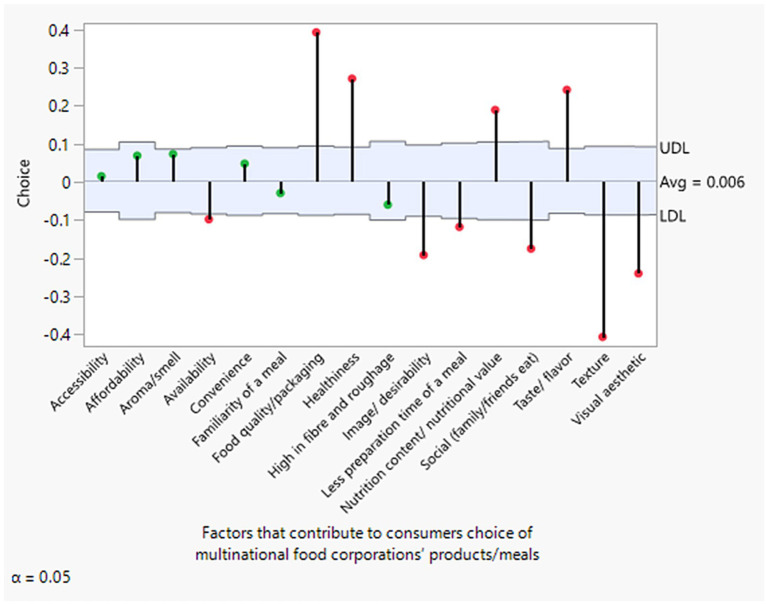
Average decision chart of factors influencing female consumers’ choices of multinational food products. Green indicates insignificant factors, while red indicates significant factors.

**Figure 4:**
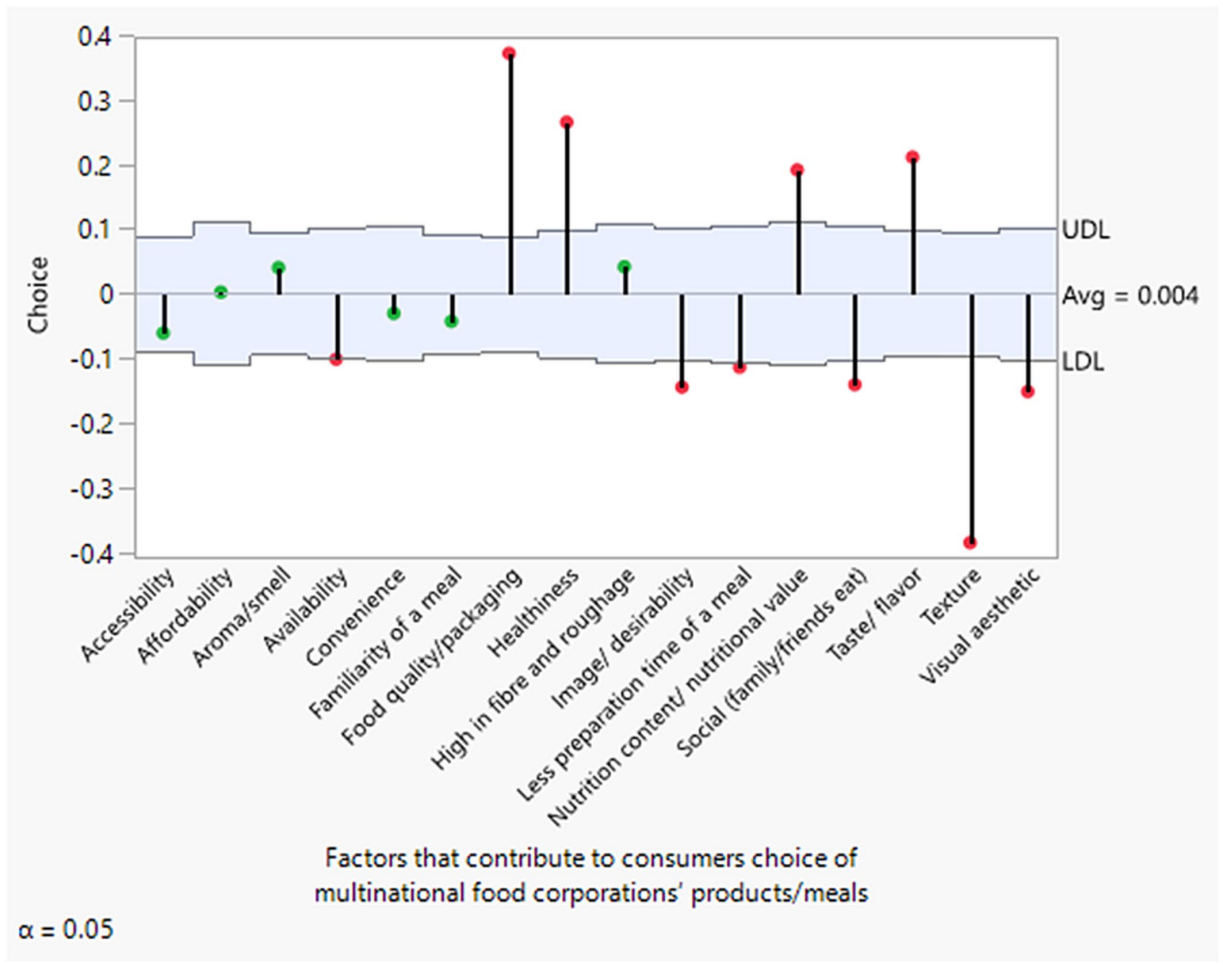
Average decision chart of factors influencing male consumers’ choices of multinational food products. Green indicates insignificant factors, while red indicates significant factors.

Figures 3, 4 display an overall average decision chart by both female and male respondents. With the exceptions of accessibility, affordability, aroma/smell, convenience, familiarity with a meal, and high fiber/roughage—which were found to be statistically insignificant for both genders—all other attributes, such as food quality/packaging, healthiness, nutritional content, and taste/flavor, show significantly positive averages and were highly valued. This is consistent with the results from all the ML models. In contrast, attributes like availability, image/desirability, meal preparation time, social factors (e.g., family/friends eating together), texture, and visual esthetics were generally less valued or exhibited negative average significant differences. These findings align with those obtained from the full dataset.

## 4 Discussion

This study integrates five machine learning (ML) models and quantitative MaxDiff designs to predict the factors influencing the meal/product preferences of urban Ghanaian consumers from multinational food corporations, as well as gender-specific differences in those preferences. Our approach is expected to be highly relevant to researchers and policymakers, as it provides a promising strategy to address the limitations of traditional statistical methods, which can suffer from diminished inference accuracy as the number of input variables and their potential associations increases. This study offers AI/ML data-driven insights and a deeper understanding of diverse consumer segments, thereby supporting the development of healthier food policies in Ghana’s changing food landscape, including tailored dietary interventions for specific gender groups. The ML models significantly outperformed the traditional statistical model. Notably, the Ridge regression model emerged as the best candidate, achieving the smallest mean RASE value, albeit at a longer fitting time. Our analysis of the full dataset revealed that all attributes significantly influenced consumer choices. In the subgroup analysis based on gender, food quality/packaging was found to be the most important factor in product or meal selection from multinational food corporations, followed by healthiness, taste/flavor, and nutritional content or value, consistent with findings from the full dataset. Although texture was statistically significant, it was consistently traded off for other attributes in both the full dataset and the gender subgroups.

Our findings highlighted the importance of food quality/packaging as the primary factor predicting meal or product choices from multinational food corporations. This aligns with results from our previous study (14), indicating a discernible pattern in the data uncovered by the ML model. As societies evolve, with more women working outside the home in both urban and rural areas and men increasingly commuting for urban and off-farm jobs, dietary habits tend to shift towards more ultra-processed foods (30). In this context, packaging or perceived food quality become crucial factors, significantly influencing consumers’ purchasing decisions (31). However, evidence shows that these ultra-processed foods are often designed with ingredients and techniques aimed at maximizing profitability. This includes features such as long shelf lives, low-cost ingredients, branded packaging, and cosmetic additives like flavorings, colorings, and sweeteners that enhance the sensory appeal of the products (32). Furthermore, the easy availability, cheapness, convenience (ready-to-consume), and quasi-addictiveness of ultra-processed foods, combined with aggressive marketing practices, contribute to the displacement of non-ultra-processed foods, including traditional cultural foods and cuisines, in people’s diets (33, 34). Therefore, it is essential to support consumers through education on the benefits of reading labels. Additionally, there should be clear promotion or regulation by the appropriate mandated agencies of food product labeling regarding their nutritional characteristics. This would ensure food transparency, providing consumers with important information about what they are ingesting (35).

We identified additional significant predictors of food preferences among urban Ghanaian consumers, compared with traditional statistical methods (14). These predictors included accessibility, affordability, convenience, familiarity with a meal, and a high fiber and roughage content. This finding provides policymakers with a deeper understanding of the factors influencing consumers’ product choices from multinational food corporations. By utilizing large datasets that encompass consumer preferences, ML algorithms can identify patterns and correlations that traditional statistical methods may overlook (36). ML has emerged as a vital tool in predicting meal choices, transforming how we understand and optimize dietary habits (37). Through iterative learning and adaptation, ML models continually refine their predictions, providing increasingly accurate insights into the complex interplay of factors influencing dietary choices (38). As a result, ML has become essential in guiding individuals toward more informed and health-conscious meal planning and consumption strategies (39).

A notable finding was the observed gender-specific differences in global food preferences. Interestingly, female respondents tended to assign greater significance to certain attributes. They valued the aroma/smell of food highly, suggesting that sensory appeal significantly influences their choices. Additionally, considerations such as affordability—ensuring that meals or products from multinational food corporations provide good value for money—convenience of ready-to-eat meals that can be bought in nearby food franchises and restaurants, accessibility, and the familiarity of certain dishes also weighed heavily on their decision-making process. In contrast, male respondents showed a stronger preference for global meals or food products that are high in fiber and roughage, recognizing the health benefits associated with these nutrients. For them, the aroma and smell of food remained important, but they also prioritized affordability and convenience in ready-to-eat meals. Understanding these distinct, gender-specific preferences is crucial for businesses. It can guide targeted marketing efforts and food product development strategies aimed at catering to diverse consumer needs, ultimately influencing dietary behaviors and promoting healthier eating habits. These insights can help nutritionists and policymakers develop targeted initiatives to enhance dietary diversity and improve nutritional outcomes (40). Comprehensive strategies are needed to implement gender-specific needs. For example, discussions around food policy should involve advisory boards or stakeholder groups that represent both men and women. Their perspectives can help shape initiatives that meet diverse needs. Additionally, policymakers should implement educational programs that focus on the nutritional requirements and preferences of different genders.

It is important to acknowledge the limitations of this study. The research focuses on specific linear ML models. However, it also paves the way for future research. Future MaxDiff designs should incorporate decision trees and ensemble models, including decision tree regression, random forest regression, gradient boosting regression, XGBoost regression, LightGBM regression, CatBoost regression, AdaBoost regression, and HistGradientBoosting regression. Additionally, these studies should involve the perspectives of stakeholders across the food industry, more male participants, and include a greater diversity in socio-economic status, while considering a wider geographical area. Future work should consider how manufacturers prioritize ethical practices and strive towards a more sustainable and equitable food system, as well as the impact of their products on society and the environment. It is crucial to recognize that the accuracy and effectiveness of suggestions derived from ML can vary depending on the quality and diversity of the underlying data, as well as the complexity of the algorithms used. More research is needed to further develop and validate these models across a broader range of demographics. Despite its limitations, the present study has several strengths. A significant strength is its novelty; this is the first study to examine the predictors of urban Ghanaian consumers’ preferences regarding meals and products from multinational food corporations using AI/ML models and a quantitative MaxDiff design. The observed gender-specific differences in the factors that influence product or meal choices offer valuable insights for tailoring dietary behavior change strategies effectively.

## 5 Conclusion

We have presented a promising approach to address challenges in statistical methods and provided comprehensive AI/ML data-driven insights into the predictors of urban Ghanaian consumers’ food preferences for products from multinational food corporations. Our study not only sheds light on the factors influencing food preferences but also contributes to the growing body of research on AI/ML applications in consumer behavior. We have demonstrated that to draw strong conclusions about global food choices, ML models must not only exhibit robust goodness of fit and superior predictions but also be interpretable and computationally efficient. By integrating multiple ML models and quantitative MaxDiff designs, we have identified additional factors overlooked by statistical methods and given policymakers a deeper understanding of the factors influencing urban Ghanaian consumers’ food preferences. This, in turn, supports the development of healthier food policies in Ghana’s evolving food landscape. The gender-specific differences suggest that dietary and food interventions may benefit from being tailored to specific populations and gender groups. Future MaxDiff designs should incorporate nonlinear and ensemble models.

## Data Availability

The raw data supporting the conclusions of this article will be made available by the authors, without undue reservation.
